# The COVID-19 Clinician Cohort (CoCCo) Study: Empirically Grounded Recommendations for Forward-Facing Psychological Care of Frontline Doctors

**DOI:** 10.3390/ijerph18189675

**Published:** 2021-09-14

**Authors:** Jo Daniels, Jenny Ingram, Anna Pease, Elaine Wainwright, Kate Beckett, Lalitha Iyadurai, Sophie Harris, Olivia Donnelly, Tom Roberts, Edward Carlton

**Affiliations:** 1 Department of Psychology, University of Bath, Bath BA2 7AY, UK; slh92@bath.ac.uk; 2 North Bristol NHS Trust, Bristol BS10 5NB, UK; Olivia.Donnelly@nbt.nhs.uk (O.D.); tomkieranroberts@gmail.com (T.R.); Ed.Carlton@nbt.nhs.uk (E.C.); 3 Centre for Academic Child Health, University of Bristol Medical School, Bristol BS8 1QU, UK; jenny.ingram@bristol.ac.uk (J.I.); a.pease@bristol.ac.uk (A.P.); 4 School of Science, Bath Spa University, Bath BA2 9BN, UK; esh26@bath.ac.uk; 5 HAS-Nursing and Midwifery, University of West England Bristol, Bristol BS16 1QY, UK; kate2.beckett@uwe.ac.uk; 6 Department of Psychiatry, University of Oxford, Oxford OX1 2JD, UK; lalitha.iyadurai@psych.ox.ac.uk; 7 Royal College of Emergency Medicine, London EC4A 1DT, UK

**Keywords:** COVID-19, frontline workers, healthcare workers, qualitative research, trauma, psychological support, occupational health, guidelines

## Abstract

This study aimed to develop empirically grounded recommendations and a coherent model of psychological care derived from the experiences and psychological care needs of COVID-19 frontline doctors, using semi-structured interviews and thematic analysis. Participants were UK frontline doctors specialising in Emergency Medicine, Anaesthetics, or Intensive Care (*n* = 31) purposively sampled for maximum variation on gender, specialty, ethnicity, and trauma-related distress; most worked in ICU during the pandemic (71%). Four themes were derived: (1) ‘coping strategies’, participants used many, including exercise, mindfulness, and “wait until it gets really bad”; (2) ‘sources of support’, participants valued embedded psychological support, digital services, and informal conversations with colleagues or family, though there was little opportunity; (3) ‘organisational influences on wellbeing’, participants reported a love–hate relationship for concepts like ‘wellbeing’, seen as important but insulting when basic workplace needs were unmet; (4) ‘improving engagement with support’, analysis suggests we must reduce physical and psychological barriers to access and encourage leaders to model psychologically supportive behaviours. Doctors’ frontline COVID-19 working experiences shine a ‘spotlight’ on pre-existing problems such as lack of physical resources and access to psychological care. Empirically grounded recommendations and a model of incremental psychological care are presented for use in clinical services.

## 1. Introduction

The psychological impact of Coronavirus Infectious Disease 2019 (COVID-19) on frontline doctors has been well documented. With up to 54% experiencing clinical levels of psychological distress [1], many report being affected by trauma symptoms, fears of contamination, moral injury, disruption of normal supportive structures, and work pressure [2,3,4,5]. These factors are associated with long-term psychological sequelae [6,7].

Guidelines to address the psychological needs of healthcare workers (HCW) amidst the COVID-19 pandemic have been developed and advocated by national professional bodies and Royal Colleges [8,9,10,11,12]; however, these resources were written at the outset of the pandemic when little was known about the impact and likely trajectory, and were not empirically underpinned or substantiated by COVID-19 specific research. We are now in a better position to develop guidelines tailored to the clinical characteristics of this group at a time when psychological support is pivotal to long-term mental health.

A recent qualitative study reported that preferences, experiences, and coping styles of COVID-19 HCW should be considered when developing “programs developed to mitigate stress” [13] (p. 1) with a recent meta-analysis indicating that more “high-quality qualitative research is urgently needed” particularly with frontline HCW whose “voices have not yet been adequately represented” [14] (p. 25). Billings et al. [14] indicate qualitative research should underpin clinical guidance, and while subsequent qualitative studies have explored frontline HCW experience of working and caring for patients during the COVID-19 pandemic [15,16,17,18,19,20,21,22] drawing out similar themes to those reported in other pandemics [14], few qualitative studies have specifically sought to develop or directly inform guidance [23]. The initial COVID-19 mental health response has recently been criticised for underutilising qualitative enquiry, overlooking lived experience, and focusing on general populations such as HCW [24]. Further qualitative research focusing on specific groups, such as doctors, underpinned and shaped by patient and public involvement, has been recommended to strengthen the COVID-19 mental health research response [24]. However, this paucity of research remains.

Doctors are likely to benefit from specialist services and interventions tailored to their needs due to the well-documented barriers they perceive to accessing psychological support [25] and the high rates of burnout and distress already prevalent in this group [26]. The unrelenting pressure of working during the COVID-19 multi-wave pandemic is only likely to have exacerbated these issues further, yet no specific care pathways or models of care have been developed to address this.

### Study Aims

The aims of this study were to develop an empirically-grounded set of recommendations and model of future-facing psychological care de-rived from the experiences of psychologically distressed doctors working on the frontline during the COVID-19 pandemic.

## 2. Materials and Methods

The COVID-19 Clinicians Cohort (CoCCo) study is a follow-on study from the ‘COVID-19 Emergency Response Assessment’, also known as CERA (IRAS: 281944) [1,27,28,29]. CERA is a five-phase longitudinal study which sought to examine the prevalence and nature of distress in frontline doctors during the first wave (Spring 2020) and second wave (Winter 2020) of the COVID-19 pandemic [1,27,28,29].

Participants in the CoCCo study were recruited from the parent CERA study participant pool. The CoCCo study has been approved by the Health Research Authority (20/HRA/6315) and University of Bath Psychology Research Ethics Committee (21-001).

### 2.1. Measures

Participants in the CERA study completed quantitative measures of psychological distress, a demographic questionnaire and further questions pertaining to personal and professional characteristics (see CERA study for further details [1,27,28]). From the CERA study battery of measures, data relating to gender, specialty, ethnicity (individual demographic items), and traumatic stress (Impact of Events Scale-Revised, IES-R) were extracted to select a purposive sample for the CoCCo study (see Section 2.2).

The IES-R [30] is a 22-item measure commonly used to measure post-traumatic distress following a pre-specified traumatic incident and has been used to evaluate the impact of infectious disease outbreaks on hospital staff [31]. The IES-R has been found to have excellent internal consistency (α = 0.96) and construct validity when correlated with the PTSD checklist (α = 0.84) [32]. The IES-R has been widely used during the COVID-19 pandemic to assess levels of trauma in frontline workers [33,34].

### 2.2. Participants

Doctors of all grades and seniority working in either the emergency department, anaesthetics or intensive care unit (ICU) in the UK and Republic of Ireland during the acceleration phase of the COVID-19 were eligible for inclusion in the parent study, CERA. Non-doctors, or those working in other specialty areas were excluded from the CERA study.

All medical specialties included in the CERA study were eligible for inclusion in the CoCCo sampling strategy due to these specialties having highest likelihood of exposure to patients with COVID-19 with consequent exposure to aerosol generating procedures (high disease exposure risks and PPE requirements). In addition, these specialties were more likely to be subject to redeployment to high intensity working environments and were the core specialties involved in frontline work, a factor predictive of psychological distress [35].

### 2.3. Procedure

In survey four of the CERA study, participants were asked to consent to be contacted for future research studies and share information. All those who indicated agreement to both formed the CoCCo sampling pool and were sent a link to study information and online consent to take part in the interview-based CoCCo study.

Of those who consented to participate, a purposive sample of doctors was selected for maximum variation on four characteristics: gender, specialty, IES-R score, and ethnicity. These factors have been associated with higher levels of traumatic stress [1,2,27,28,33,34,35]. Those with IES-R scores 24 or above (indicating clinical levels of distress) were eligible to take part, as these doctors were more likely to have experience of psychological distress and demonstrate care needs.

The interviews sought to elicit an understanding of perceived psychological needs and preferences in relation to psychological care; the topic guide was informed by scope of study aims, the current evidence base and input from the PPI group. The guide consisted of four sections: describing experiences on the frontline; identified psychological needs and support already accessed; preferences relating to psychological care (past/present/future); positive experiences of the pandemic. The latter served as a psychological buffer for potentially difficult elements of the interview, and to offer the opportunity to explore other relevant aspects through a non-problem focus.

Semi-structured interviews using the topic guide were conducted by telephone or video call by five trained qualitative researchers (AP, JI, EW, JD, LI) with health services research, clinical, and health psychology backgrounds. Participants with high IES-R scores were interviewed by clinical psychologists in the team. At the end of the interview participants were debriefed about the study and sent information on sources of support.

Interviews were recorded using encrypted devices and stored securely and separately from personally identifiable data. All data are stored in accordance with the University of Bath Data Security and Confidentiality Policy and the Data Protection Act 2018.

### 2.4. Analysis

Framework analysis was applied to the data, following the seven steps outlined in Gale et al. [36] including a rapid analysis of main themes (concurrent with data collection) prior to detailed thematic analysis [37], as described in Table 1. The rapid analysis form was developed using the interview topic guide to capture the main points from each interview and to aid thematic analysis. Interviews were audio recorded, transcribed verbatim, and anonymised by a professional transcription service. Transcripts were checked for accuracy and coding facilitated using NVivo 12 Pro (QSR International Pty Ltd. and thematic analysis applied to the transcripts by AP and KB. Interviews continued until data saturation was achieved, in that no new themes were being generated from the data.

Ten transcripts (32%) were double coded to improve robustness of the analysis and minor discrepancies resolved through discussion—15% of transcripts were recoded blind by a second coder and high agreement between codes was reached, supporting the validity of the results. All coding and recoding was discussed between qualitative team members, debating minor differences until consensus was achieved.

All analytical decisions were shared and discussed by the qualitative research group using a consensus process to agree the final coding and thematic framework and reported in accordance with the COnsolidated criteria for REporting Qualitative research (COREQ) reporting checklist [38].

### 2.5. Development of Recommendations and Model of Psychological Care

The identified themes derived from interview data formed the basis of an initial set of recommendations and illustrative model of psychological care, which was developed by the research team through an iterative process. The recommendations, model of care, and a summary document of themes and quotes were then distributed to the PPI panel who were then invited to comment on the face validity, salience and personal relevance of the recommendations. All members of the PPI committee provided individual input via email correspondence, and once all feedback had been received, the recommendations and model were adapted and consensus agreement gained from the PPI group for the amended version. The refined versions of the recommendations and model, and summary document of themes and quotes were then distributed to the expert advisory group (EAG) for consultation via email, with feedback invited on the clinical relevance, broader context, and practical implications of the recommendations. Once amended, members of the EAG and PPI approved the final version.

The EAG comprised senior clinicians and researchers in the field representing Health Protection Research, Psychiatry, Emergency Medicine, Clinical Psychology, Intensive Care, Occupational Medicine and Staff Wellbeing.

### 2.6. Patient and Public Involvement

A PPI panel comprising six frontline doctors of different specialties, seniority, ethnicity and gender were appointed to advise on all aspects of the study, including research design and recommendation development. All PPI members were offered renumeration for their time.

## 3. Results

Of the 346 CERA [1,27,28] participants who gave consent to be contacted for follow-on studies, 44 consented to interview (12.7%), and 36 were contacted to take part, as per our sampling frame requirements. Five people who consented were contacted for an interview but did not respond.

Thirty-one interviews were conducted with doctors (age range 27–59) from 26 different hospital Trusts across England and Wales. Interviewee characteristics are shown in Table 2. Twenty-two (71%) doctors (all but one of the anaesthetists) were redeployed to or worked in an intensive care unit (ICU) during the pandemic. IES–R scores ranged from 24–74 (mean 43.8, SD 13.3). Interview length ranged from 16 to 68 min (mean 41 min).

### 3.1. Main Themes

Analyses generated six key themes: ‘accounts of challenging frontline situations’, ‘impact on staff’, ‘coping strategies’, ‘sources of support’, ‘organisational influences on wellbeing’, and ‘improving engagement with support’. The first two themes mirror findings reported extensively elsewhere [15,16,17,18,19,20,21,22], making no novel contribution to knowledge in the field. In this paper, we report only the latter four themes (Table 3), which are aligned with our study aims, and underpin development of recommendations and a model of care to support clinicians’ wellbeing. This is important, as our interviews showed many of the challenges described—such as excessive workload, fatigue, and burnout—existed before the pandemic. By illuminating and heightening these entrenched issues, the COVID pandemic shone a spotlight on the need for change.

*“In some ways maybe having had this peak is a perfect opportunity to incorporate more into the workplace, because clearly it took until breaking point for us to even acknowledge that we have an endemic problem within the profession.”*

(#115, F, Intensive Care)

*“There’s already a significant degree of burnout and exhaustion, and I think that one of the worst things about this pandemic was that it was on top of that; a lot of people were already working by running on empty almost and then this happened.”*

(I#121, M, Anaesthetist, ICU)

Themes are presented with illustrative numbered quotes in Table 4, Table 5, Table 6 and Table 7; participants are identified by gender (F, M), specialty (Emergency Medicine EM, Intensive Care Medicine ICM, Anaesthetics An), and whether they had experience of working in the ICU.

#### 3.1.1. Coping Strategies

Participants described using a range of strategies to cope with the current circumstances including exercise, getting outside, being more open with feelings, mindfulness, and journaling. Exercise was a common strategy, which could be related to participants’ stated need to ‘keep busy’ to cope with what they were going through (A1). Journaling was mentioned by two participants who found this was useful as a way of offloading thoughts and experiences. Being more open with feelings was mentioned as a way of engaging with others and reducing isolation. Other participants reported drinking alcohol, smoking, and eating as their way of coping (A2).

More specific psychological coping strategies included ‘storing things up’ and ‘waiting until things got really bad’, and believing that psychological support was more needed by others than themselves.

*Storing things up:* Many participants described feeling compelled to keep going during the height of the pandemic; they felt they could not take time to process events so stored things up in a box for later. Some viewed this as a positive strategy (A3). Clinicians also described feeling disconnected from the emotions they would usually have towards very ill or dying patients. Others expressed fear for what would happen following the current peak of the pandemic and questioned whether they would be able to cope.

*Wait until it gets really bad:* Some participants described having insight around when to access psychological support, but if it was a limited resource, felt they should only use it with a clear and definite ‘need’. Others described how they felt unable to tell when it was time to ask for support and that, traditionally, doctors were not good at this (A4).

*Psychological help is for others not me:* There were several comments that clinicians were better at recognising and attending to others’ needs than their own, and set aside their own needs if others needed support more (A5).

**Table 4:** Illustrative quotes for Coping Strategies theme.

Quote Number	Illustrative Quote
A1: Exercise	*A1: “I would rather go for a run or see my family, or bake, or do something creative rather than sit and breathe quietly with my eyes shut.” (#106, F, An, ICU)*
A2: Drinking	*A2: “To be perfectly honest with you more drinking, not to stupid quantities where I feel like I’ve needed to go and get help, but to quantities where I wouldn’t want… when I have children I wouldn’t want to be drinking as much as I am drinking now.” (#127, M, EM, ICU)*
A3: Store it up.	*A3: “There’s been a lot of stuff I’ve seen that hasn’t been very pleasant, and obviously in the moment you think about it and you’re upset about it, but I have somewhat just packaged that up and put it to the one side.” (#103, F, An, ICU)*
A4: Wait until it’s bad.	*A4: “I found it very easy to just think to myself I’ve just got to get on with this, this is it’s normal to be feeling really anxious and to not be sleeping. I know that burnout is a concept that exists but that can’t be happening to me.” (#122, M, An, ICU)*
A5: Psychological help is for others	*A5: “I am fine,.. I don’t need that service as much as other people, so I’m not going to use that resource. I don’t want to be the one who is taking up a resource unnecessarily and wasting somebody’s time.” (#109, F, EM)*

#### 3.1.2. Sources of Support

Participants described a range of formal and informal sources available to them, either at work or outside, some of which were more beneficial than others. Participants’ perceptions of sources of support are described as ‘what helped’ and ‘what didn’t help’.

**What helped**

*Talking to colleagues:* There were strong and consistent descriptions of the value of support from colleagues during the pandemic, with descriptions of affection for team-mates and co-workers, and the support they had given and received during intensely stressful times. Being able to take time out, share concerns, and discuss the challenges with colleagues (especially senior ones) was universally seen as beneficial. It helped to unpack what had happened and to allay anxiety. Participants found this informal support helpful both in terms of acknowledging experience but also exploring strategies (B1). Often this support was the most valuable and had the biggest positive impact on their ability to cope. They missed post-shift informal breakfasts or trips to the pub and opportunities to ‘decompress’.

*Embedded psychological therapists:* Participants described embedded psychological support services available during the pandemic as positive, though rarely used. They liked knowing that someone was there if they needed them and valued their availability and continued presence as it removed some of the barriers to access; but availability could be an issue (B2).

*Accessing specialist support:* While very few participants reported accessing formal psychological support through work, those that did found it helpful. Some participants had already contacted counsellors and psychologists outside work prior to the pandemic and resumed or continued this contact. This was helpful in setting their experience in a wider context and normalising their reactions (B3).

*Apps and phone services:* Some participants reported using mental wellbeing apps, telephone support services, and phone messaging app groups promoted by their Trusts or organised within clinical groups, and some found them valuable for managing ongoing mental wellbeing. Participants were mostly aware of what was available but did not always engage with it (B4).

*Friends and family:* Informal positive support, including friends and family, was described by many participants as very important, especially from partners. Friends were an important outlet for feelings and distraction but talking about work was only useful if they were also medics, so the complex medical context was understood.

**What did not help**

*No time to attend or to talk:* There were frequently barriers to accessing more formal support at work, including the appropriateness of sharing emotional difficulties with other colleagues and the ability to get away (B5). While peer-to-peer and senior colleague support could be extremely beneficial, some participants felt that these interactions were not regarded as a good use of their time (B6).

**Table 5:** Illustrative quotes for Sources of Support theme.

Quote Number	Illustrative Quote
B1: Talking to colleagues	*B1: “We’re a really good team, so just making sure that everybody is feeling okay, and actually talking about our experiences, … share those experiences that have been difficult, I think that really helps. […] being in a good team has to be the winning thing really.” (#114, F, ICM)*
B2: Embedded support	*B2: “you see the psychologists are just normal people, getting a tea in the tearoom. They are really accessible, and then when people start crying at work they just sidle up and say, “Are you okay?” And I think that’s really good.” (#103, F, An, ICU)*
B3: Specialist support	*B3: “I’ve had two sessions with her [psychologist] just over the phone and that’s been quite good. Working in acute specialties, you are at the pointy end of quite a lot of drama and quite a lot of situations which might stick with you and impact on your mental health and actually maybe you need a little bit of time to process, and it’s helpful to talk through.” (#107, F, An, ICU)*
B4: Resources	*B4: “Our Trust has an excellent wellbeing resource page, … plus everywhere you look now there’s guidance on resilience and wellbeing.” (#105, F, An, ICU)*
B5: No time to attend…	*B5: “Most of the time on my shift I can’t just drop out for things, if you manage to time your break for that time you can do it, if you don’t then you can’t, or you will just end up being tied up in a complex case that you can’t walk away from. In that sense they were quite inaccessible to me.” (#109, F, EM)*
B6: ... or to talk.	*B6: “Often at work if you talk for a few minutes it’s like, “Right get on now,” and there’s not really any time … and we’re policed constantly. It’s always like you’re on a time limit, you never really get to sit down and have that chat, and we can’t see each other outside of work.” (#110, F, EM)*

### 3.1.3. Organisational Influences on Wellbeing

Broader external factors which either supported or impeded clinicians’ wellbeing included organisational structures, values and attitudes, and responses to differences between COVID waves. These themes contextualise the personal and clinical experience within wider organisational factors.

*Positive influences:* The concept of wellness or wellbeing activities at work came up fairly frequently. Most people were aware of services or activities provided through work under the label of ‘wellness’, including resilience training, meditation, or organised sports. Participants invariably had a love/hate relationship with this concept—some found it useful and supportive, and others found it meaningless, irritating, and insulting. Those that appreciated the support often connected this service to an overall ethos of caring about staff and their mental wellbeing (C1).

*“It is the little things”:* When asked about what support has been most helpful, all participants identified small gestures of kindness or changes to the work environment that made a significant impact on their wellbeing. This was often connected to a feeling of being cared about, looked after, valued, or making being at work easier or more comfortable. Small things seen as crucial to proper functioning included being able to meet basic human needs for hot food and drink, comfortable chairs, rest areas, walls with pictures or supportive messages, free parking, plentiful scrubs, and working hot showers (C2).

*Negative influences:* There were many examples of bad communication, poor leadership, and accounts of the impact of government and press handling of the pandemic that left participants feeling frustrated and angry. While some people reported that their Trust was supportive and helpful, others were left feeling undermined, threatened, and let down (C3).

*Resilience:* A significant number of participants commented on the futility of being offered guides to healthy eating and resilience by their Trusts as this missed the point and was insulting. They would be looking after themselves if they could, but the demands of their situation prevented them from doing so and these initiatives failed to address the underlying causes of stress. Resilience was not something they considered they lacked (C4, C5).

*COVID timeline:* Generally, participants felt that despite the uncertainty and newness of the first wave in March 2020, it was in many ways easier to manage than what came afterwards (C6). The relentlessness, lack of preparedness, and changing patient demographic in the second wave in winter 2020 was harder on staff (C7).

**Table 6:** Illustrative quotes for Organisational Influences on Wellbeing theme.

Quote Number	Illustrative Quote
C1: Positive influences	*C1: “We had loads of things around the hospital like decompression rooms or quiet rooms where you just go and be quiet, and they would put colouring books and coffee machines there so you could sit and reflect on what had happened, which was really helpful.” (#102, F, An, ICU)*
C2: The little things	*C2: “I think a huge thing about morale in the NHS actually it is the small things, …the things that really get people down or really lift people’s spirits are not very big, it’s free tea and coffee. There are no rest facilities for doctors anymore, so if there’s a chair that pulls out and a blanket or a pillow, that really lifts people’s spirits. It’s really little stuff like that.” (#103, F, An, ICU)*
C3: Negative influences	*C3: “It felt at times very vulnerable and just a little bit maybe sacrificial you didn’t have any control over anything really, you were just given what you were given and had to work with it, and it would constantly change, and I think it felt like there was a lack of respect from people at managerial and senior levels as to what we were actually doing.” (#107, F, An, ICU)*
C4: Resilience	*C4: “If you say resilience to a doctor, … you’ve lost them already… someone wants to [talk to] me about resilience and they have not just done the week that I have just done, walk a week in my shoes and then talk to me about resilience.” (#105, F, An, ICU)*
C5: Resilience	*C5: “It’s just I think the word resilient should never be used, because it’s just become a swear word,… you’re upset about the fact that you can’t manage your childcare, and your shift, and your pay has been cut… what you need is some resilience training [laughs]. Just makes us all so angry.” (#104, F, An)*
C6: COVID 1st wave	*C6: “The first wave with the redeployment of staff we had lots of staff,…. we worked 24 h, we had packs of teams that worked together, so we did feel like we had enough staff.” (#111, F, EM)*
C7: COVID 2nd wave	*C7: “I felt like this time we had months to prepare, and actually when it arrived it was bigger than was anticipated, we were totally overwhelmed, and people were not redeployed up until the 11th hour.” (#115, F, ICM)*

### 3.1.4. Improving Engagement with Support

**Facilitators and treatment preferences**

*Access and support options:* Opinions on how psychological support should be accessed included online, face-to-face, one-to-one and in a group, and as more formal access to safe spaces to talk. One-to-one support was often suggested because of a need for safety of information and trust. Group sessions were felt to be useful if with colleagues; small groups were favoured over large ones to maximise how much each person could contribute. Online sessions were understandable in the context of the pandemic, but most preferred face-to-face if available. They valued talking therapies in various formats (D1, D2).

*Supporting emotional wellbeing:* Participants felt a system for supporting emotional needs would be helpful, either as brief clinical reflective sessions at the end of a shift or through increasing awareness and asking about colleagues’ wellbeing (D3). Some participants felt that if support was built into inductions or made more visible within the hospital setting, then more people would access it. Teaching clinicians about the signs of emotional distress and when/how to get support would also be beneficial.

*Someone who understands:* Many participants felt quite strongly that talking with someone who had been through similar experiences would be important or preferred. This would remove the need to describe in detail or relive the distressing things they had dealt with, and there would be an awareness of what they had experienced (D4).

*Embedded psychological support:* Although very few participants had availed themselves of embedded psychological support (i.e., a psychologist working within the service), it was generally regarded as a favourable model which could help in normalising clinicians’ reactions to their exposure to trauma (D5).

*Anonymity/independence:* For some participants, the independence of support was important, and they would rather engage with services that were not connected to the workplace. This would remove worry about stigma and concern that colleagues, managers, or superiors would judge them negatively for having accessed support (D6). It was also connected to feeling safe, to get the most out of any support accessed, and to explore their feelings in an honest way (D7).

*Leadership:* Participants ranged from trainees to consultants and most mentioned how leaders can influence engagement with support services. Examples of good leadership, including modelling and demonstrating care about the mental wellbeing of oneself and one’s staff, improved morale overall (D8).

*Normalising psychological reactions:* Participants were clear that there needed to be measures to make it acceptable to reveal and discuss psychological reactions to the challenges they faced at work and that leaders, education, and embedded psychologists could start this conversation (D9). However, there was also a feeling that this was changing, and a few gave examples of how normalising discussions about mental health had helped to make access easier and less daunting.

**Barriers to access**

*Timing and not knowing what is available:* The biggest barrier to accessibility was timing—shift work and long hours made finding the time to attend support sessions more challenging (D10). Some participants expressed wanting to access support once the current surge was over. Also knowing what support was available and which would suit best was another problem, especially when clinicians were already overwhelmed with information (D11).

*Stigma, culture, and consequences:* The stigma associated with seeking support for mental wellbeing was cited as a barrier to engaging with available support by many. There was some recognition that this stigma was slowly changing, but there were several clear examples where participants or others had been shamed for accessing support. They described how seeking support might be seen as a sign of weakness, and how medics are often required to ‘put up and shut up’ when it comes to their own mental wellbeing. This was combined with concern of the impact on their future career (D12, D13, D14).

**Table 7:** Illustrative quotes for Improving Engagement theme.

Quote Number	Illustrative Quote
D1: Safe place	*D1* *: “The concept of a safe space where you can take timeout that’s actually recognised as timeout is absolutely something that should exist.” (#130, F, An, ICU)*
D2: With colleagues	*D2* *: “I think it would be helpful to be with colleagues, I find that shared experience and the people around you, like the team that supported you through it, having them around for the aftercare is quite helpful.” (#113, F, ICM)*
D3: Clinical debrief	*D3:* *“There would probably be scope for something more proactive, so for example a structured debrief at the end of every shift.” (#122, M, An, ICU)*
D4: Someone who understands	*D4* *: “I think it is trust that you can just say how you feel, and also the fact that they would understand how you felt, because they understand it, because they have been through it, which really helps.” (#102, F, An, ICU)*
D5: Embedded support	*D5:* *“* *It’s just the visibility of it is important because it normalises it… it’s okay to not be okay, … there’s not necessarily anything wrong with you or anything that needs treating. It’s just you have seen something horrible and you want to have a cry about it. So I think the real presence and visibility of that kind of [psychologist] support is really useful.” (#103, F, An, ICU)*
D6: Anonymity	*D6* *: “I know some people have deliberately avoided going through work in case there’s any stigma attached to that when it comes to annual review or anything like that,” (#124, M, An, ICU)*
D7: Safety of information	*D7* *: “Have a blanket measure to help everybody, a safe place to talk, knowing that if I say something somebody is not going to come back to me and say “you said this, why did you say it?”” (#129, M, EM)*
D8: Leadership	*D8* *: “People who I admire clinically and professionally also trying to step up and actually look after our welfare themselves as individuals and taking on a little bit more of a welfare role, that’s been nice, when I am sure they themselves have actually been experiencing all the same things I am experiencing.” (#107, F, An, ICU)*
D9: Normalise psychological reactions	*D9* *: “Sometimes I want to be able to say something like I felt like this, and it’s awful I felt like this, but I did feel like that, why?” (#127, M, EM, ICU)*
D10: Timing	*D10* *: “How are we supposed to get protected time in the context of the NHS being under unprecedented pressure, to do things that are good for our wellbeing? (#112, F, EM)*
D11: What’s available?	*D11* *: “It feels a bit like the support is there, but you need to go looking for it, as opposed to being encouraged to actively engage with it, I think.” (#122, M, An, ICU)*
D12: Stigma	*D12* *: “I feel like there’s a fair amount of stigma about mental health, about mental wellbeing, and admitting if you’re struggling or finding things difficult, and I don’t know if I would have gone for fear of being stigmatised for it.” (#109, F, EM)*
D13: Culture	*D13* *: “It is just a toxic culture within medicine … which comes from the top down … It makes speaking up about having difficulties very difficult …within that acute specialties umbrella there’s almost a macho.… it’s a very masculine thing of heroism… but there’s something there about people think that they should be somehow superhuman and not [affected] in a normal way by some of the very abnormal things that we’re involved with.” (#117, F, EM, ICU)*
D14: Consequences	*D14:* *“I understand why they are not accessing the mental health support services. There’s so many reasons, one is the perception of failure if you have a mental health problem, second is you don’t want your employer to know if you’re struggling in any way because they are helping your progression to your endpoint of being a consultant, no one wants to admit failure as a doctor.” (#105, F, An, ICU)*

## 4. Recommendations and Model of Psychological Care

As detailed in Section 2.5, the qualitative data and identified themes derived from the data formed the basis for an initial set of recommendations and model of psychological care, which was then iteratively refined and shaped by the PPI panel and EAG until consensus was achieved. Amendments and adaptations suggested by the PPI and EAG were minor and broadly related to practical implications and wording adjustments (e.g., suggestions regarding the use of a ‘welcome pack’ to raise mental health issues; recommended evidence-based interventions, e.g., TRiM [39]; wording of particular recommendations such as ‘clinical reflective spaces’). No substantial deviations were made from the original recommendations. Box 1 reports the final set of recommendations developed to underpin the future care and support needs of doctors working on the frontline, encompassing basic needs of sustenance, information and communication, embedded support, and psychological interventions.

Box 1 **Recommendations for psychological care of frontline doctors.**

**(1)**  **Basic needs and physical resources for all staff**

**Figure 1:**
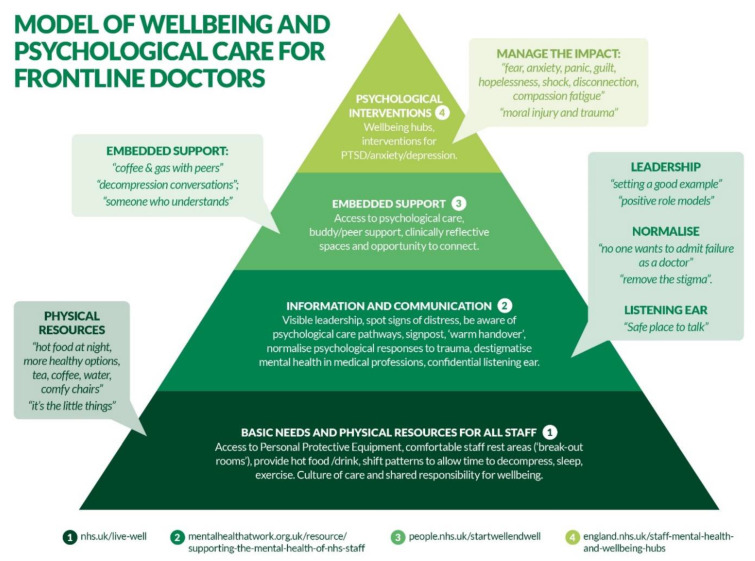
Model of wellbeing and psychological care for frontline doctors.

Sustenance (provision of both hot and cold food and drinks), rest, and sleep should be addressed and reflected in local guidelines and shift patterns to account for the need to rest and recharge between shifts for all staff (Figure 1. step 1). Working contract allowance for rest periods should be honoured and exercised by all staff grades and specialities. Designated quiet staff rest or ‘decompression’ areas should be provided and protected for all staff. Basic safety needs should be addressed through adequate provision of PPE.

A culture of care and shared responsibility for staff wellbeing should be actively promoted and facilitated by all clinical leaders. Staff should feel safe to speak up about their wellbeing without fear of repercussion and should be made to feel that their wellbeing is important to peers and clinical leaders. Key to psychological safety is strong role modelling from senior leaders who should regard discussion about wellbeing or acts to improve wellbeing in self or others as positive action to be encouraged, acting with humility, showing genuine care, and helping to foster a strong sense of team belonging. This should be supported through access to confidential spaces to reflect and seek collegiate or senior support.

**(2)**  **Information and communication**

Normalising experiences: frontline healthcare work is intrinsically stressful, and being affected by difficult or traumatic cases is a normal response; this should be acknowledged and regularly discussed by peers and leaders. Being able to distinguish between ‘normal’ reactions and the emergence of more substantial difficulties is also key; with time, normal distress begins to dissipate, rather than intensify or impact daily functioning. Psychological impact of recurrent traumatic incidents on staff should be openly discussed, with particular focus on de-stigmatising views about mental health within the medical workforce.

Basic information regarding warning signs of deteriorating mental health in self and others should be offered to all staff (including clinical leaders), promoting shared responsibility for colleague wellbeing. Signs might include heightened emotional responses, absenteeism, withdrawal, and sleep disruption. Raising awareness of mental health issues should take place within medical training and routinely at a local level through practical ‘welcome packs’, which communicate trust ethos and attitudes towards mental health, pathways of support, and information regarding local lines of reporting. Local Trusts may wish to recommend specific resources such as eModules on trauma awareness and information on Trust-wide staff wellbeing teams, which could guide discussion.

Signposting availability of resources and what to access is useful even in the absence of psychological difficulties. Information relating to sources of support should be displayed and regularly refreshed in clinical areas, provided within standard operating procedures and as part of local guidance (e.g., return to work documentation). ‘Warm handovers’ should be used to facilitate transition to psychological care where possible, promoting likelihood of uptake.

**(3)**  **Embedded support**

Face-to-face or video call clinical ‘reflective spaces’ between peers/senior staff should be offered and embedded within clinical teams. This may be ad-hoc or organised reflective spaces such as Schwartz rounds. More formal peer-to-peer interventions for structured support should be available; this may be in the form of Trauma Risk Management (TRiM) or ‘StartWell > EndWell’ procedures. These can be particularly helpful in time-poor contexts and should be encouraged and facilitated by clinical leads or trained health professionals.

‘Psychologically safe’ spaces which foster constructive reflection on own/others practice and on the moral dilemmas and challenges of health care delivery should be made available to all. These conversations should be dealt with confidentially and sensitively, including follow-up options to discuss with external peers or mentors; informal support seeking must be encouraged by clinical leaders to facilitate this.

**(4)**  **Psychological interventions**

Pathways to appropriate level care are imperative: a stepped pathway of psychological care responsive to the presenting need of the individual should be offered. This might include provision to ‘step up’ from self-help/self-management approaches (such as use of apps, meditation, or bibliotherapy), to formal peer support and, at the highest level referral to high-intensity interventions, should this be required (e.g., CBT for PTSD). Referral outside the immediate team/serice to occupational health to address workplace issues, ‘well-being’ hubs or mental health services should also be part of a complete local pathway, which should outline route from peer or embedded support to mental health interventions and services.

Psychological interventions: should be evidence-based and tailored according to presenting difficulties. Support should be:

- embedded within services (in reach) where possible, offering access to hard to reach groups;
- suitable for ongoing/repeated traumatic events;
- sensitive to mental health stigma known in this population;
- tailored to individual need but considering the wider context of the team wellbeing;
- available in different formats e.g., group, individual, online;
- provided on a regular and reliable basis reflecting best practice;
- accessible to shift workers e.g., by release to attend during work hours;
- able to account for the unique characteristics of working on the frontline, such as moral distress, dealing with uncertainty, fear of harm and concerns over person wellbeing;

On review, the final recommendations (Box 1) and illustrative model were closely aligned with the model of care, as set out by the British Psychological Society (BPS) ‘Psychological needs of healthcare staff document’ [9], which highlights the proportionate incremental demand with each step of a hierarchy of wellbeing. This model specifies the importance of meeting ‘basic needs’ for all and ‘psychological intervention’ for the few who are likely to need it; the model offers ‘stepping up’ the hierarchy to more specialized interventions when required. With the permission of the BPS and collaboration with lead author of the document [9] (JH), the BPS model of psychological care was further populated and refined and using empirical evidence generated from this study (see Figure 1) in order to provide an illustrative model of psychological care based on the recommendations derived from this study. All ‘steps’ of the BPS model were retained (with minor title changes); however, more comphrensive details from the recommendations were provided in each ‘step’, with accompanying quotes from the qualitative data to highlight doctors’ voiced concerns in their own words.

## 5. Discussion

This study aimed to develop empirically grounded recommendations and a future facing model of psychological care for frontline doctors, derived from the experiences of those most psychological distressed in the EM, An, and ICU specialties. Findings identified four key themes, including the range of strategies used to cope with their psychological stress; who they found most useful in helping them; their love–hate relationship for concepts like ‘wellbeing’; and how physical and psychological barriers to access must be reduced and leaders encouraged to model psychologically supportive behaviours. These themes were used to underpin the development of recommendations (see Box 1) and a model of psychological care (see Figure 1), shaped by PPI input and expert advice. Our findings offer further insight into the ongoing psychological impact of COVID-19 and principally, the unmet needs which resonate with longstanding unresolved issues, specifically workplace culture, mental health stigma, and neglect of basic physical needs such as adequate food, rest, and sleep. Frontline healthcare work is and always has been, both intrinsically stressful and rewarding [25]. However, the nature of the occupational climate plays a pivotal role in this; working on the frontline during COVID-19 has cast a ‘spotlight’ on working practices within the NHS, drawing attention to pre-existing problems. These problems now urgently need to be addressed and can no longer be ignored.

In relation to previous studies, findings from this study reflect similar themes drawn out elsewhere during the pandemic [15,16,17,18,19,20,21,22] suggesting ‘commonality’ in experiences of frontline workers [40]. This extends across specialties, gender and ethnicity within our sample—few discrepancies were identified between groups, despite recent quantitative studies reporting significant differences in psychological distress related to age, gender [2,3,33,34,35], ethnicity, and specialty [1,27,28]. Accounts of working through two waves of the pandemic reflected a sense of immense and prolonged pressure, the second wave causing considerably higher stress. Those experiencing higher levels of distress reported finding it difficult to manage without their usual coping mechanisms, instead using alcohol and food; strategies associated with poorer psychological outcomes during the pandemic [41,42]. Most commonly raised issues were concerned with basic sustenance and comfortable spaces to ‘decompress’. The need for adequate access to hot food and time to reflect and process feelings are issues that predate COVID-19 [43] but have been reported repeatedly throughout the pandemic [8,9,10,11,23]. Despite calls to address this issue, it remains unresolved—getting the basics right is an essential foundation to wellbeing and necessary psychological care.

Consistent with current research [16,44] and guidance [8,9,10,11,12], ‘peer support’ in the workplace was valued, yet time pressure and organisational processes prohibited this being accessed fully. This suggests problems in organisational and local provision (e.g., insufficient staff/capacity) to facilitate the need to ‘rest and digest’, use appropriate clinical reflective spaces [45], or similar ad-hoc/organised sessions. Top-down organisational support and formal operationalising of local support structures are needed; working contracts enable this yet cultural expectations appear to prohibit it. The introduction of more formal peer to peer support is routinely facilitated, such as Schwartz Round [46], Trauma Risk Management (TRiM) [39], and StartWell > EndWell’ [47] psychologically informed procedures; however, this must form part of a coherent care pathway, which enables doctors to access the help they need at the time and in the form they need, honouring contractual breaks in work shifts and signposting that involves a ‘warm handover’, i.e., direct facilitation of access to care [48].

Due to the complex and unique occupational demands of doctors, adaptations are required to promote and enable appropriate access to psychological care [25]. This is particularly related to mental health stigma reported in the profession acting as a barrier to engaging with services [25,49], but also relates to accessibility of services due to shift work and time pressure. This issue has again been further exacerbated by COVID-19 and the perceived absence of support to access care, such as the expectation that mental health related appointments should be attended during ‘time off’, as reflected in our findings and elsewhere [22]. Although participants were aware and positively regarded psychological support embedded in hospital settings, few accessed or engaged with the services available to them. Standard mental health services in their current form are unlikely to be sufficiently tailored to the specific challenges faced by frontline workers; however, there is a strong evidence base for structured psychological interventions [50,51] with work ongoing to adapt and refine for this group [52].

This study produced empirically grounded recommendations specifically focused on psychological care beyond the pandemic. The recommendations are drawn from the needs and preferences of frontline doctors in this study, and identify structures, processes, and procedures that facilitate delivery of care at each level. One key barrier identified has been the absence of a clear pathway or coherent framework within which to mobilise or deliver interventions, despite an abundance of research-based recommendations and professional body guidance [8,9,10,11,12]; few COVID-19 mental health guidance documents propose how recommendations fit together to produce a pathway of care.

It is further suggested that these recommendations and model of psychological care are implemented for use with frontline workers and in the recently developed ‘wellbeing hubs’.

### 5.1. Strengths and Limitations

This study derived specific, practical recommendations for care from interviews with doctors following two waves of the COVID-19 pandemic. In response to calls to action [24], this study sought to draw on both the experience of those on the frontline but also a PPI group to assess salience, validity, and relevance of recommendations. A panel of field experts were also consulted to ensure recommendations were relevant and feasible. A purposive sample based on ethnicity, gender, specialty, and seniority were used in this study, increasing confidence in our findings and their relevance to these groups.

This study focuses solely on doctors rather than healthcare workers (HCW) more broadly: it is erroneous to assume that doctors, nurses, and other HCW are a homogenous group. Different professional groups are likely to have unique experiences working on the frontline and have been found to differ in their preferred coping mechanisms [44]. Finally, this study sampled only those experiencing clinical levels of psychological distress; while the rationale supports this, we are not able to offer insight into the needs of those who have not been psychologically impacted, and indeed, why that might be.

### 5.2. Future Research Directions

Interventions such as trauma-focused Cognitive Behavioural Therapy (CBT) and Eye Movement Desensitisation Reprocessing (EMDR) are effective for treating traumatic stress from exposure to a past trauma and are relevant to doctors, with research ongoing to support this. However, psychological interventions suitable for exposure to repeated or ongoing trauma, as experienced by healthcare staff, are lacking [50]. There is a need for effective interventions that are brief, repeatable, and low-intensity; that can be easily used each time a new trauma occurs; or that provide primary prevention (e.g., pre-emptive training to manage the impact of traumatic events when they occur).

## 6. Conclusions

Empirically grounded recommendations and an incremental model of psychological care were derived from psychologically distressed doctors’ accounts of frontline working during COVID-19. Encompassing both basic needs such as lack of physical resources and access to specialist psychological care for those most in need, these recommendations and model of care must be implemented at an organisational level and led by clinical leaders who are well-supported, confident, and competent in delivering the necessary changes to address psychological wellbeing of doctors.

## Figures and Tables

**Table 1:** Framework analysis steps.

Stage 1.	Transcription: Audio recordings were transcribed verbatim and pseudonymised. Detailed notes were taken by each interviewer (all authors), structured around the topic guide and questions to produce a rapid coding matrix.
Stage 2.	Familiarisation with the interview: Two authors familiarised themselves with the interview by reviewing the rapid coding notes and full transcript.
Stage 3.	Coding: AP developed a matrix in NVivo 12. The initial coding process involved systematically reading (and re-reading) the rapid coding notes and full transcripts for each participant, assigning data to relevant question headings and identifying key subthemes within each component.
Stage 4.	Developing a working analytical framework: Qualitative team met to discuss in detail the findings as enabled by the rapid analysis matrix, to agree on the key themes.
Stage 5.	Applying the analytical framework: All transcripts were imported into NVivo 12 and the analytical framework established. Each transcript was coded by systematically assigning data to a node in the analytical framework. The framework was revisited after 20 transcripts and additional sub-codes created to aid differentiation of distinct meanings emerging within themes. Ten transcripts were double coded by three researchers (EW, KB, JI)
Stage 6.	Charting data: Drawing on the full analysis in NVivo, AP, and KB created a table of the key themes with illustrative quotes and reviewed it with all authors.
Stage 7.	Interpreting the data: During regular team meetings (10 meetings over the analysis phase), and via circulation of written materials with the Clinical Advisory Group, impressions and interpretations of the data, coding, and the analytical framework were discussed and agreed. This process was ongoing throughout the analysis process.

**Table 2:** Characteristics of the doctors interviewed.

Characteristic (*n* = 31)	*n* (%)
Specialty	Anaesthetics = 14 (45%) Emergency Medicine = 13 (42%) Intensive Care = 4 (13%)
Gender	Female = 19 (61%) Male = 12 (39%)
Seniority	Consultant or equivalent = 10 (32%) Middle grade doctor = 14 (45%) Junior doctor = 7 (22%)
Ethnic Origin	White = 23 (74%) Black and Minority Ethnic background = 8 (26%)
IES-R Score	Range 24–74; Mean 43.7 (SD 13.3)

**Table 3:** Themes and subthemes.

Theme	Sub-themes	Includes
A. Coping strategies. Positive and less positive coping strategies	(1) Storing things up (2) Wait until it gets really bad (3) Psychological help is for others not me	(1) Coping by disconnecting (2) Not realising how bad things have become (3) Others might benefit/need support more
B. Sources of support. Formal and informal support available.	(1) What helped at work and outside work (2) What didn’t help at work and outside work	(1) Peer-peer support, senior support, embedded psychological therapists, clinical debrief, apps, family and friends. (2) Peer-peer, senior informal contact, no time to access support.
C. Organisational influences on wellbeing. Factors which supported or impeded wellbeing.	(1) Positive influence: Organisational (2) Negative influence: Thoughts on ‘resilience’	(1) Environmental changes and ‘the little things’, managerial support. (2) Negative environmental changes had a big impact, poor managerial decisions.
D. Improving engagement with support. Psychological treatment preferences identified by clinicians.	(1) Facilitators and treatment preferences. (2) Barriers to access	(1) Embedded psychological support, someone who understands us, trust and anonymity, leadership role models, normalising psychological reactions, confidentiality of what is shared. (2) No time off, loss of trust, lack of signposting, too much information, stigma, culture and consequences

## Data Availability

Qualitative data from the CoCCo study is available on request by contacting J.D. on jd494@bath.ac.uk; due to the distressing nature of the data and accounts of experiences on the frontline, this is not currently being made publicly available.
